# Hybrid poplar plantations are suitable habitat for reintroduced forest herbs with conservation status

**DOI:** 10.1186/2193-1801-2-507

**Published:** 2013-10-04

**Authors:** Kathleen Boothroyd-Roberts, Daniel Gagnon, Benoit Truax

**Affiliations:** Centre d’étude de la forêt, Université du Québec à Montréal, CP 8888 succ. Centre-ville, Montreal, Quebec H3C 3P8 Canada; Fiducie de recherche sur la forêt des Cantons-de-l’Est/Eastern Townships Forest Research Trust, 1 rue Principale, Saint-Benoît-du-Lac, Quebec J0B 2M0 Canada; Department of Biology, University of Regina, 3737 Wascana Parkway, Regina, Saskatchewan S4S 0A2 Canada

**Keywords:** Understory, Abandoned farmland, Forest corridors, Non-timber forest product, *Asarum canadense*, *Sanguinaria canadensis*

## Abstract

Plantations of fast-growing tree species may be of use in conservation by accelerating the restoration of forest habitat on abandoned farmland and increasing connectivity in fragmented landscapes. The objective of this study was to determine if hybrid poplar plantations can be suitable habitats for the reintroduction of native forest plant species and, if so, which abiotic factors predict successful reintroduction. Four species of forest herb species (*Trillium grandiflorum*, *Sanguinaria canadensis*, *Maianthemum racemosum, Asarum canadense*), of which three have legal conservation status, were transplanted into experimental plantations of two hybrid poplar clones and nearby second-growth woodlots at six sites in southern Quebec, Canada. The transplanted individuals were protected from deer browsing with exclusion cages. After two years, the plant responses of all four species were stable or increased over two years in both types of hybrid poplar plantations. *Sanguinaria* showed a better response in the plantations than in the woodlots, preferring the rich post-agricultural soils of the plantations with low C:N ratios. *Asarum* and *Maianthemum* showed no significant difference between stand types, while *Trillium* grew better in the woodlots than in the plantations. Much of the variability in the response of the latter three species was unexplained by the measured environmental variables. These results suggest that certain forest herb species can be reintroduced as juvenile plants into plantations, knowing that their spontaneous recolonization is often limited by dispersal and/or seedling establishment. Plantations could also contribute to the conservation of biodiversity by providing an environment for the cultivation of forest herb species as an alternative to their destructive harvest from natural populations.

## Background

Deforestation for agriculture in North America and Europe has resulted in the loss and fragmentation of forest habitats in many landscapes (e.g., Bélanger and Grenier 2002; Peterken 2000). More recently, the concentration and intensification of agriculture has resulted in the abandonment of less productive agricultural land, which is now in various stages of old field succession (Domon and Bouchard 2007; Le Houérou 1993; Poyatos et al. 2003; Roura-Pascual et al. 2005). The process of abandonment has been gradual, with the more marginal sites generally being abandoned first, and since reconverting to mature secondary forest (Domon and Bouchard 2007; Glitzenstein et al. 1990), while other, richer sites tended to be kept in production or in pasture until more recently and are currently at earlier stages of succession.

The afforestation of abandoned farmland could contribute to the conservation of biodiversity by restoring forest habitat and increasing connectivity in fragmented landscapes (Brockerhoff et al. 2008). Plantations on abandoned farmland could restore certain ecological functions and components of forest plant diversity, while at the same time increasing the economic value of these lands through wood production (Fortier et al. 2012; Brunet et al., 2012). While there is debate over the ecological impacts of plantations, recent reviews suggests that plantations established on degraded or agricultural lands, rather than replacing natural ecosystems, tend to promote biodiversity (Bremer and Farley 2010; Brockerhoff et al. 2008). Fast-growing tree species such as hybrid poplars, commonly planted in North America and Europe, could be especially useful in such projects, creating a closed tree cover from an open field in the span of one to two decades (Marchand and Masse 2007; Truax et al. 2012). Hybrid poplar plantations could function as transition stands in the restoration of deciduous forest environments on old fields, by acting as nurse stands (Boothroyd-Roberts et al. 2013) or through subsequent under-planting with native tree species (Truax et al. 2012; Gardiner et al. 2004).

While a plantation can create a tree cover on an old field very rapidly, it is important from a conservation perspective to understand whether the resulting environment can be suitable for native plant species adapted to forest understories. Forest herbs are of particular interest, since many are sensitive to disturbance and slow to recolonize after the abandonment of agriculture (Flinn and Vellend, 2005). Older hybrid poplar plantations in Europe support populations of certain forest herb species (Endels et al. 2004), but young (10 years or less) hybrid poplar plantations are often devoid of most forest herb species typical of natural forests (Boothroyd-Roberts et al. 2013, Soo et al. 2009). Plantation understories may differ from those of natural secondary forests as a result of site preparation disturbances, differences in tree species composition, reduced tree species richness, and simplified stand structure (Aubin et al. 2008). However, Boothroyd-Roberts et al. (2013) have shown that hybrid poplar plantations can produce shade and leaf litter in quantities similar to those of natural second-growth forests and can serve as nurse stands for native tree species. It is not clear whether the absence of forest herb species in plantations is due to dispersal limitation or habitat constraints. Many Eastern North American forest herb species are known to be very slow dispersers (Whigham 2004) and the presence or absence of these species may not accurately reflect the habitat value of a plantation. Experimental reintroductions are useful for directly testing the suitability of an environment for forest herb species (De Keersmaeker et al. 2004; Singleton et al. 2001; Flinn and Vellend 2005). This approach can provide a synthetic measurement of the various abiotic and biotic factors that combine to affect habitat quality, overcoming the limitations of studying existing plant communities, often strongly influenced by dispersal. Baeten et al. (2009) used this approach to test the performance of four native understory species in hybrid poplar plantations in Belgium. They found after 8 years that two of the species had declined over time in the plantations, but not in old-growth forests, an effect attributed to increased competition within the plantations. The other two forest species performed equally well in the plantations as in the old-growth forests.

Plantations also have the potential to assist conservation efforts by creating environments suitable for the cultivation of forest herb species of horticultural or medicinal value to replace harvesting from the wild. Many forest herb species are slow-growing and their harvest from wild populations is usually unsustainable (Charron and Gagnon 1991; Nantel et al. 1996; Nault and Gagnon 1993). Several understory species, including white trillium (*Trillium grandiflorum*), wild ginger (*Asarum canadense*), and bloodroot (*Sanguinaria canadensis*), are already listed as vulnerable in Quebec, due to risks of overexploitation of wild populations (MDDEP 2010). Their cultivation in plantation understories as non-timber forest products (NTFPs) could provide a sustainable supply of these plants, as well as an additional and earlier return on investment for plantation owners (but see findings of Burkhart and Jacobson 2009).

The present study used experimental reintroductions as an assessment of the habitat value of hybrid poplar plantations. Four species of native understory herbs, three with legal conservation status in Quebec, were transplanted as juveniles or adults to simulate their reintroduction in a restoration context or their cultivation as NTFPs. We compare 10-year-old hybrid poplar plantations with second-growth forests of natural origin, which is the most common habitat type for understory herbs in agro-forested landscapes in eastern North America. We hypothesized that even if the understories of young hybrid poplar plantations differ from natural forest understories in terms of certain characteristics, the shading and leaf litter provided by the hybrid poplars would be sufficient to reduce competition from ruderal species and allow native understory herbs to survive and grow. We also compare plantations of two hybrid poplar clones with different parent species. Previous studies have found differences among species and hybrids in terms of productivity on different soil types (Truax et al. 2012), branching patterns and leaf biomass (Fortier et al. 2010), root density (Fischer et al. 2006), nitrogen cycling (Schimel et al. 1998), shading, understory vegetation cover (Boothroyd-Roberts et al. 2013) and understory vegetation biomass (Fortier et al. 2011). In a previous study at the same sites as the present study, we found that understory communities were more similar to those of natural woodlots in plantations of a *Populus maximowiczii* and *P. balsamifera* (M × B) hybrid than a *P. deltoides* and *P. nigra* (D × N) hybrid (Boothroyd-Roberts et al. 2013), and therefore we hypothesized that reintroduced plants may survive and grow better in the M × B plantations.

The questions we attempt to answer with this study are: (1) Can understory herbs be reintroduced successfully in hybrid poplar plantations? (2) Can the reintroduction be as successful in hybrid poplar plantations as in second-growth woodlots typical of the study region? (3) Is reintroduction success different under different poplar hybrids? (4) How do abiotic factors influence reintroduction success?

## Results

### General results of transplant experiment

Four forest herb species were transplanted into plantations of two hybrid poplar clones and into woodlots. The four herb species varied considerably in initial size. None of the *Trillium grandiflorum* or *Maianthemum racemosum* plants was at reproductive maturity and 72% of *Trillium* plants were seedlings with only one leaf. *Maianthemum* plants had between one and 7 leaves each (3.34 ± 1.23; mean ± s.d.). *Sanguinaria canadensis* plants were somewhat larger at the time of transplanting; many showed evidence of having flowered earlier that spring, although flowers were not counted for year 1. *Sanguinaria* was planted as clumps with an initial size of one to four leaves (1.40 ± 0.74). *Asarum canadense* plants were the largest of the four species and all clumps contained mature, flowering individuals (10.6 ± 5.0).

In the spring of year 3 (two years after transplanting), average survival rates by plot (all stand types combined) were 61% ± 33% (mean ± s.d.) for *Asarum*, 52% ± 32% for *Maianthemum*, 77% ± 29% for *Sanguinaria*, and 59% ± 29% for *Trillium.* Of the surviving plants, 67% of *Asarum* plants, 77% of *Sanguinaria* and 12% of *Maianthemum* plants produced flowers. Of the surviving *Trillium* plants, 94% had reached the three-leaf adult stage and 18% flowered. Damage from slugs and other invertebrates was evident in many plots for all of these species, although this damage was not quantified. Important differences in biomass were visible among the six study sites. In several plots at the HAM and CAT sites, only a few small plants remained in the spring of year 3 while at the BED site, the lowest elevation site with rich bottomland soil, all four species were flourishing.

**Figure 1:**
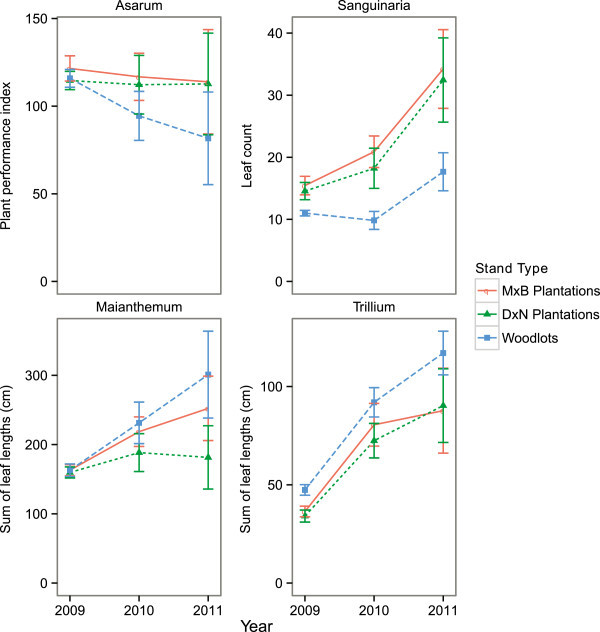
**Changes in responses of four species of forest herbs in the two years after transplantation.** Titles above figure panels indicate the species of forest herb (Asarum: *Asarum canadense*; Sanguinaria: *Sanguinaria canadensis*; Maianthemum: *Maianthemum racemosum*; Trillium: *Trillium grandiflorum*). Forest herbs were transplanted into three stand types (MxB plantations: plantations of a hybrid of *Populus maximowiczii* and *P. balsamifera*; DxN plantations: plantations of a hybrid of *P. deltoides* and *P. nigra*; woodlots: second-growth hardwood woodlots). Error bars indicate standard error of the mean.

The average number of leaves and flowers of *Asarum* (as a proxy for biomass) remained approximately constant in the plantations and tended to decline in the woodlots over the two years after transplantation (Figure 1). The number of leaves of *Sanguinaria* and the sum of the leaf lengths of *Trillium* tended to increase over the two years after transplantation in all three stand types, while the sum of leaf lengths of *Maianthemum* tended to increase in the MxB poplar plantations and the woodlots, but not in the DxN poplar plantations. For *Maianthemum* and *Trillium*, the increase tended to be greater in the woodlots while the reverse was true for *Sanguinaria*. For all species, the variability among plots increased over the two years, reflecting a differential response to the different sites (Figure 1).

### Effects of site and stand type

The results of ANOVAs show that there tended to be greater differences in the responses of the transplanted plants among sites than among stand types (Table 1). Site had the largest effect on all variables, except for the number of *Sanguinaria* leaves and flowers. Interaction effects were significant only for number of *Asarum* leaves and flowers and for the sum of *Trillium* leaf lengths (p < 0.05).

**Table 1:** **Fixed effects of ANOVAs on responses of four species of transplanted forest herbs**

Variable	Site		Stand type^c^		Interaction	
	F	p	F	p	F	p
*Asarum canadense*						
Number of leaves + flowers per plot^a^	43.3888	<.0001 ***	2.3344	0.1148	3.7802	0.0038 **
*Maianthemum racemosum*						
Total leaf length per plot^a^	4.0108	0.0104 *	2.2950	0.1188	1.5807	0.1739
Number of flowers per plot^a^	11.4558	<.0001 ***	2.4167	0.1070	0.7774	0.6256
*Sanguinaria canadensis*						
Number of leaves + flowers per plot^b^	0.3094	0.5883	15.8166	0.0004 ***	1.7179	0.2207
*Trillium grandiflorum*						
Total leaf length per plot^b^	61.5570	<.0001 ***	6.2614	0.0137 *	27.6855	<.0001 ***
Number of flowers per plot^b^	3.1470	0.1014	0.9703	0.4068	0.0579	0.9440

^a^Data from 5 sites were included in ANOVA.

^b^Data from 2 sites were included in ANOVA.

^c^Stand type: plantations of two clones of hybrid poplar and second-growth hardwood woodlots.

*Significance at p < 0.05.

** Significance at p < 0.01.

*** Significance at p < 0.001.

Stand type had a significant effect on number of *Sanguinaria* leaves and flowers, with more leaves and flowers in both types of poplar plantations than in the woodlots (Table 2). There was also a trend toward a better *Asarum* response in both types of plantations than in the woodlots, although this is mostly attributable to the exceptional response in the BED site plantations (but not the BED woodlots), in which *Asarum* produced about two to four times the number of leaves and flowers than at other sites. The sum of *Trillium* leaf lengths was significantly higher in the woodlots than in either type of poplar plantation, largely a result of the very poor survival in the plantations at the BRO site (but not the BRO woodlots). There was a trend toward better *Maianthemum* responses in the woodlots than in the DxN poplar plantation. No significant differences were detected between the two poplar clones (Table 2).

**Table 2:** **Response measurements and effects of stand-type (p-values) of four species of transplanted forest herbs**

Variable	Hybrid poplar plantation						
	DxN clone^a^		MxB clone^b^		Second-growth hardwood woodlot		p-value of environment effect^c^
	Mean ± s.e.	n	Mean ± s.e.	n	Mean ± s.e.	n	
*Asarum canadense*							
Number of leaves + number of flowers per plot	114.20 ± 34.56	15	120.47 ± 35.39	15	81.64 ± 26.41	15	0.1148
*Maianthemum racemosum*							
Total leaf length per plot	1733 ± 538	15	2472 ± 551	15	3010 ± 627	15	0.1188
Number of flowers per plot	0.40 ± 0.21	15	0.93 ± 0.38	15	1.04 ± 0.35	15	0.1070
*Sanguinaria canadensis*							
Number of leaves + number of flowers per plot	73.33 ± 9.78 (a)	6	70.17 ± 7.74 (a)	6	26.50 ± 4.31 (b)	6	0.0004 ***
*Trillium grandiflorum*							
Total leaf length per plot	861 ± 276 (b)	6	940 ± 306 (b)	6	1171 ± 111 (a)	6	0.0137 *
Number of flowers per plot	1.17 ± 0.31	6	1.00 ± 0.63	6	1.83 ± 0.54	6	0.4068

^a^DxN clone: a hybrid of *Populus deltoides* and *P. nigra.*

^b^MxB clone: a hybrid of *Populus maximowiczii* and *P. balsamifera.*

^c^p-values are from ANOVAs with site and stand-type as fixed effects (see text for details).

^*^Significance at p < 0.05.

^***^ Significance at p < 0.001.

Letters following response measurements indicate significant differences between environments at p < 0.05 in Tukey HSD means comparisons tests.

### Response of transplants to environmental variables

Environmental variables were quite heterogeneous among the six sites and among stand types. At the most productive plantation sites, the plantations were similar to the woodlots in terms of light availability and leaf litter biomass, while at the less productive sites, the plantations provided much less shading and litter than the woodlots. The woodlot soils were on average more humid and more acidic than those of the poplar plantations, with higher C contents, higher C:N ratios, and higher concentrations of N, K, and Mg. However, the degree of difference in soil properties between the woodlots and the plantation/field environments were not consistent across all sites and at some sites the relationships were opposite those of the general trends (for detailed results of these environmental variables see Boothroyd-Roberts et al. 2013).

The effects of these environmental variables on understory herbs were explored using linear mixed-effects regression and model selection, the results of which are presented in Table 3. June soil moisture was the most important variable for predicting the response of *Asarum,* followed by soil Ca, and these two variables were included in the best model (with the lowest AICc), *Asarum* had more leaves and flowers in drier plots with higher soil Ca. Together, June soil moisture and soil Ca explained an estimated 31% of the variability in *Asarum* response. Elevation was not important for *Asarum,* since the plant response was similar at the highest-elevation site and at the 2nd lowest-elevation site.

**Table 3:** **Regression coefficients and relative importance of environmental variables predicting responses of forest herb species**

		Environmental variable								
Response variable	Statistic	Elevation	June soil moisture	Soil C:N ratio	Soil Ca	Soil Mg	Soil P	Leaf litter biomass	Diffuse light	Marginal R^2^of best model*
*Asarum:* Number of leaves and flowers per plot	Best model coefficients		−0.43		0.20					0.31
	Multi-model average coefficients	−0.22	−0.41	−0.08	0.20	−0.01	0.08	0.01	−0.10	
	Importance	0.14	**1.00**	0.17	**0.66**	0.11	0.13	0.10	0.31	
*Sanguinaria:* Number of leaves and flowers per plot	Best model coefficients	−0.61		−0.56						0.60
	Multi-model average coefficients	−0.61	−0.09	−0.55	−0.07	−0.06	0.11	−0.03	−0.01	
	Importance	**0.74**	0.11	**1.00**	0.07	0.06	0.15	0.06	0.05	
*Maianthemum:* Sum of leaf lengths per plot	Best model coefficients		−0.33		0.33	−0.37			−0.49	0.36
	Multi-model average coefficients	−0.18	−0.35	0.20	0.36	−0.40	0.03	−0.15	−0.49	
	Importance	0.19	**0.94**	0.31	**0.88**	**0.90**	0.10	0.15	**1.00**	
*Maianthemum:* Number of plants flowering	Best model coefficients	−0.56							−0.26	0.41
	Multi-model average coefficients	−0.55	−0.13	−0.10	−0.07	−0.09	0.01	−0.10	−0.29	
	Importance	**1.00**	0.17	0.22	0.06	0.15	0.07	0.18	**1.00**	
*Trillium:* Sum of leaf lengths per plot	Best model coefficients			0.30	−0.66					0.19
	Multi-model average coefficients	−0.43	0.13	0.30	−0.61	−0.41	−0.02	−0.15	−0.03	
	Importance	0.03	0.09	**0.53**	**0.78**	0.22	0.07	0.12	0.08	
*Trillium:* Number of plants flowering	Best model coefficients							−0.43		0.16
	Multi-model average coefficients		−0.02	0.09	0.07	−0.02	−0.19	−0.44	−0.18	
	Importance		0.05	0.10	0.11	0.10	0.15	**0.67**	0.08	

* Represents the proportion of the total variance in the data explained by the fixed effects (Nakagawa and Schielzeth 2013).

Importance values > 0.5 are indicated in bold.

The *Sanguinaria* response was predicted by elevation and soil C:N ratio in the best model, explaining an estimated 60% of the response variability, and these were also the two variables with the highest importance values. *Sanguinaria* tended to have more leaves and flowers at lower elevation sites with lower soil C:N ratios. In particular, *Sanguinaria* had few leaves and flowers at the highest elevation site, LAP. Because of missing data, this site (LAP) was excluded from the ANOVA and the remaining two lower-elevation sites did not differ significantly in terms of *Sanguinaria* response. However, the six hybrid poplar plantation plots from the LAP site, our highest elevation site, were included in the regression analyses.

For *Maianthemum*, the availability of diffuse light was the most important environmental variable predicting the sum of leaf lengths (a proxy for biomass), and was also important for predicting flowering, with a better response in shadier conditions. June soil moisture (negative effect), soil Ca (positive effect) and soil Mg (negative effect) were also important for predicting the sum of leaf lengths and were included in the best model. However, these three soil variables were relatively unimportant for predicting flowering. Flowering was instead influenced by elevation. The environmental predictors in the best models explained an estimated 36% of the variability in total leaf lengths and 41% of the variability in flowering.

The response of *Trillium* was less easily predicted by the environmental variables measured in this study, with the best models explaining only approximately 19% of the variability in total leaf lengths and 16% of the variability in flowering. Soil Ca (negative effect) had the highest importance value in predicting total leaf length and was the only variable included in the best model, but its importance value (0.78) was much lower than the highest values calculated for the other three species (1.0 for the most important variables), indicating a higher degree of uncertainty. Flowering was predicted by leaf litter biomass (negative effect) in the best model. This variable also had the highest importance value; however, this importance value (0.67) was lower than those calculated for the other response variables.

## Discussion

### Reintroduction success

Two years after transplantation, all four species showed a capacity for survival and growth in hybrid poplar plantations. *Sanguinaria* plants had grown better in the hybrid poplar plantations than in the woodlots, while the opposite was true for *Trillium* plants. *Asarum* and *Maianthemum* plants showed no significant difference between stand types, although *Asarum* tended to respond with better growth in the plantations and *Maianthemum* tended to respond with better growth in the woodlots. The experimental introductions showed that site conditions are important for transplantation success, since all four species showed high mortality in certain plots. In the most suitable hybrid poplar plantations, *Sanguinaria* and *Asarum* showed a capacity for rapid clonal growth.

The four species each responded somewhat differently to the environments, suggesting different niches. *Sanguinaria* grew better at lower elevations, which is to be expected since this species is near the northern edge of its range in the study area (Kiger 1997). However, neither *Asarum* nor *Trillium* showed an obvious preference along the elevation gradient, which is unexpected since these species are also near the northern edge of their ranges in the study area (Case 2002; Whittemore et al. 1997), and the LAP site is actually outside of the natural range of *Trillium grandiflorum* (Québec, Ministère du Développement durable, de l’Environnement et des Parcs 2005). Another unexpected result is that *Maianthemum* was more likely to flower at lower elevations, despite the fact that it is a more northerly distributed species than the other three (LaFrankie 2002).

Shading was an important factor for *Maianthemum,* which showed poorer responses in less shaded environments. This may be explained by the increased pressure of competition from other understory species that were reinvading the transplantation plots in the less-shaded stands. At our sites, differences in canopy openness among the plantations were highly correlated with understory vegetation cover, which consisted mostly of shade-intolerant species (Boothroyd-Roberts et al. 2013). In agreement with our results, competition was an important factor in a transplant experiment in hybrid poplar plantations in Belgium (Baeten et al. 2009). We speculate that *Asarum* and *Sanguinaria* were less vulnerable to competition due to their larger initial size. In the case of *Trillium*, although it may have been vulnerable due to its small initial size, it was only planted in a subset of three relatively well-shaded sites, whereas *Maianthemum* was planted in all six sites, including those with less developed plantation canopies and understories with higher herbaceous total cover.

Soil properties were important for all species. *Asarum* and *Maianthemum* grew better in well-drained soils with high concentrations of Ca (and high pH). *Maianthemum* also grew better in soils with less Mg. *Sanguinaria*, on the other hand, responded more strongly to the C:N ratio, growing better in low C:N ratio soils. These results are unsurprising, given that all three of these species typically grow in rich sugar maple forests (Gagnon and Bouchard 1981; St-Jacques and Gagnon 1988; Gauthier and Gagnon 1990; Frère Marie-Victorin 1995; Lamoureux 2002).

These habitat associations help to explain the better performance of *Asarum* and *Sanguinaria* in the plantations compared to the woodlots. Three of the hybrid poplar plantations (at the BED, BRO and LAP sites) were located on rich, well-drained post-agricultural soils. *Asarum* grew particularly well in these three plantations, while *Sanguinaria* grew well at the BED and BRO plantations, located at lower elevations. In contrast, the woodlots in this study tended to have more poorly-drained, acidic soils with higher C:N ratios (Boothroyd-Roberts et al. 2013). These characteristics are probably typical of forested areas in many post-agricultural landscapes, in which lands considered marginal for agriculture were more likely to remain forested or to be abandoned earlier (Foster 1992; Flinn et al. 2005, Domon and Bouchard 2007).

*Trillium grandiflorum* is also typically described as growing in “rich” environments (Case 2002; Québec, Ministère du Développement durable, de l’Environnement et des Parcs Québec, Ministère du Développement durable, de l’Environnement et des Parcs ; Québec, Ministère du Développement durable, de l’Environnement et des Parcs 2005; Lamoureux 2002); however, in the present study, Ca was weakly negatively correlated with *Trillium* growth. This result may be due to confounding effects from other, unmeasured variables. The better performance of *Trillium* in the woodlots, compared to the plantations, cannot therefore be explained by the present dataset.

Much of the variability observed in this study in the responses of *Asarum*, *Maianthemum* and *Trillium* was not explained by any of the measured environmental variables, indicating that other, unmeasured variables played an important role. For *Trillium* and *Maianthemum*, their smaller initial size, which is believed to be the largest single factor in transplant shock (Vasseur and Gagnon 1994), would have made them more vulnerable to meteorological conditions and small herbivore damage. Although deer (a factor excluded in this study) are generally the most important herbivores for forest understory species (Whigham 2004), the effect of small herbivores, particularly slugs, was potentially important, as noted anecdotally in this study and in a previous study on *Asarum* in southern Ontario (Liang and Stehlik 2009). Below-ground herbivores can also play an unobserved role in mortality or in limiting growth (Hunter 2001). Herbivory may explain the weak correlations of *Asarum, Maianthemum* and *Trillium* response with the measured environmental variables in this study. Also, we speculate that the very poor survival of *Trillium* in the BRO plantations may have been due to the negative effect of the higher clay content in this plantation (the only site with a clay loam; Table 4) during the dry summers, although this hypothesis could not be tested with the present dataset.

**Table 4:** **Characteristics of the six study sites in the Eastern Townships region of Quebec, Canada**

			Plantation		Woodlot	
Plantation sites	Elevation (m)	Temperature: Mean	Estim. age since	Soil textural	Minimum stand	Dominant canopy species
(abbreviations)		annual (°C)	abandonment (yrs)	class	age (yrs)	
Bedford (BED)	80	6.0	12	Sandy loam	29 - 54	*Acer rubrum, Fraxinus americana*
Brompton (BRO)	170	5.3	5 - 10	Clay loam	34	*F. americana*
Ste-Catherine (CAT)	230	5.3	25	Loam	21 - 42	*A. rubrum, F. americana, Quercus rubra*
Ogden (OGD)	260	5.1	25	Loam	39	*A. rubrum, Betula papyrifera, Thuja occidentalis*
Ham (HAM)	320	4.0	25	Loam	38	*B. alleghaniensis, A. saccharum*
La Patrie (LAP)	440	3.7	12	Silty loam	58	*A. saccharum*

### Ecological implications

The success after two years of the transplants in the hybrid poplar plantations in this study is the first evidence for the suitability of certain hybrid poplar plantation environments for these four species of forest understory plants. The differential response of the four species to the different environmental variables underlines the importance of selecting understory species for reintroduction that are adapted to the conditions at the site of interest. These results support other findings (Boothroyd-Roberts et al. 2013; Truax et al. 2012; Gardiner et al. 2004) that hybrid poplar stands could function as transition stands in the restoration of deciduous forest environments on old fields.

Understory herbaceous plants could be transplanted into plantations as was done in this study and given a chance to establish while benefiting from protection from large herbivores and an initial reduction of understory competitors. Protection from herbivory is critical, as an over-abundant population of whitetail deer (*Odocoileus virginianus*) can seriously impair restoration efforts of understory herb populations (Lubbers and Lechowicz 1989; Ruhren and Handel 2003; Whigham 2004). This model of forest restoration would be especially useful for the rapid creation of habitat corridors to link existing woodlots across agro-forested landscapes. Sites should be chosen to correspond as closely as possible to the natural habitats of the understory species, in terms of elevation, drainage and soil type. Success is more likely in plantations that have attained a high level of canopy closure.

This study also provides evidence that the major limitation to the natural colonization of plantations and secondary forests by understory plants is in the dispersal and/or seedling emergence or establishment stages, rather than in the suitability of the habitats for mature plants. Indeed, many Eastern North American forest herb species are known to be very slow dispersers (Whigham 2004), such as *Trillium*, *Sanguinaria* and *Asarum*, whose seeds are likely dispersed by ants. Post-dispersal seed predation can also limit recruitment (Bruun et al. 2010), and environmental stresses can affect seedling emergence and establishment stages (e.g., Albrecht and McCarthy 2009; Bierzychudek 1982; Nault and Gagnon 1993; Vasseur and Gagnon 1994). Litter could also affect seedling recruitment, either positively by increasing soil moisture or negatively by acting as a physical barrier (Albrecht and McCarthy 2009). However, once juveniles or mature individuals are transplanted successfully into these sites, most deciduous forest understory species in north-eastern North America can propagate clonally and can eventually spread within a site without needing to produce seed. Long-term studies with more species are needed to validate these conclusions. The creation of a central database of reintroduction projects would also help to validate these conclusions since many reintroduction experiments are carried out without the results ever being published (Godefroid and Vanderborght 2011).

One factor that may be more important in a longer-term study is the effects of competition on clonal growth. In particular, below-ground competition from the hybrid poplar trees may have an increasing effect once the tree roots re-expand after being partly removed from the planting area prior to transplanting. The M×B hybrid poplar clone had a much denser root system than the D×N clone (K. Boothroyd-Roberts, field observations), the effects of which could create a difference in survival and growth of understory plants between the two plantation types in later years. It has also been suggested that secondary chemicals in *Populus balsamifera* litter can reduce nitrogen availability for other plant species (Schimel et al. 1998), and the M×B poplar clone, with this species as a parent, may also have such an effect. Different meteorological conditions in later years may also expose vulnerabilities of the transplanted plants in one stand type or another. For example, the difference in measured soil moisture between the plantations and the woodlots may become more critical during a severe summer drought or a more pronounced wet season.

### Implications for non-timber forest product cultivation

The results of this study also suggest that it would be ecologically feasible to cultivate forest herb species in plantations such as the hybrid poplar plantations in this study, as an alternative to their cultivation in naturally regenerated woodlots, but especially to their destructive harvest from natural populations. Three of the transplanted species (*Asarum*, *Sanguinaria*, and *Trillium*) are officially designated as vulnerable in Quebec because of the threat that harvesting whole plants from natural populations represents.

More research is needed to evaluate the economic feasibility of this type of system; however, a study by Burkhart and Jacobson (2009) found that growing herbaceous NTFPs under natural forest canopies was not economically viable, except for ginseng (*Panax quinquefolius*) and goldenseal (*Hydrastis canadensis*). Economic feasibility would depend on an assessment of the long-term productivity of these plants on the transplantation sites, as well as the initial costs of the plant material. Several authors have suggested that transplanting adult plants is more likely to be successful than planting seeds, since seedlings and juveniles are generally more vulnerable to adverse environmental factors than are mature plants (Mottl et al. 2006; Ruhren and Handel 2003; Vasseur and Gagnon 1994). However, planting larger plants also carries a greater initial cost than planting from seedlings or seed. Economic feasibility may be increased by combining NTFP production with wood-production in plantations. Partial tree harvests can be carried out in winter when a snow cover protects understory plants; however, a more detailed assessment is necessary to evaluate the compatibility of these two types of production.

## Conclusions

This study shows that it is possible for forest understory plants to survive and grow under a hybrid poplar plantation that only ten years earlier was an open field. This result is one piece of evidence in support of the possibility of using hybrid poplars, or more generally, fast-growing hardwood trees, to quickly recreate an understory-like environment on an abandoned field that can then be used for ecological restoration or NTFP production.

Although the results obtained may not be generalizable everywhere, they are probably applicable to many regions of northeastern North America. It is likely that many landowners in this region could achieve similar results on abandoned farmland on their properties. For a landowner interested in practicing forestry, cultivating non-timber forest products, or restoring a high-value forest ecosystem, the afforestation of an abandoned field may potentially be a more interesting option than using existing woodlots, which on many properties are on poorly drained land or land of otherwise little value. This option becomes especially useful at lower elevations, where land-use pressure from agriculture is stronger and existing woodlots are rarer and more likely to be unsuitable for the restoration of rich hardwood forests. The lower elevations are also where hybrid poplars can grow the most rapidly and where the restoration of a closed canopy is entirely feasible within a decade.

## Methods

### Study area and plantation sites

**Figure 2:**
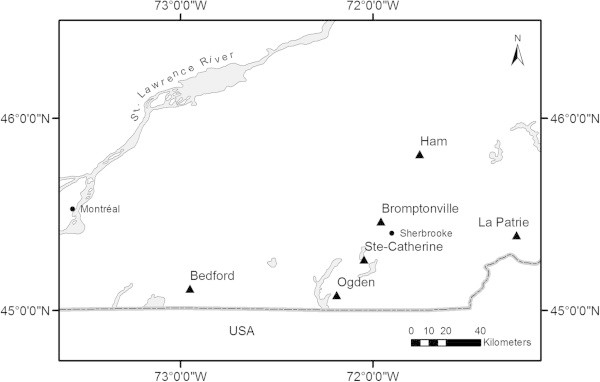
**Study sites in the Eastern Townships region of Quebec, Canada.** Study sites are indicated by triangle symbols (▲).

This study took place in the Eastern Townships region of Quebec, Canada (Figure 2), across a regional climatic gradient corresponding to an elevation gradient from the St. Lawrence Lowlands to the foothills of the Appalachian Mountains. This gradient spans three ecological regions: region1a-T, at the lowest elevations and with the mildest climate, with sugar maple and hickory (*Acer saccharum - Carya cordiformis*) mature forest stands typical of mesic sites; region 2c-T, at mid-elevations, characterized by sugar maple - basswood (*A. saccharum - Tilia americana*) stands; and finally region 3d-M, at the highest elevations, characterized by sugar maple - yellow birch (*A. saccharum - Betula alleghaniensis*) stands. The landscapes of southern Quebec represent an excellent opportunity to study the multi-functional potential of tree plantations. Many regions have been largely deforested in the past due to agriculture; however, agricultural activities have since been largely concentrated into the St. Lawrence Lowlands, resulting in the abandonment of many former fields outside this region (Domon and Bouchard, 2007).

The present study uses a series of similar experimental hybrid poplar plantations, planted in May 2000 for a study on hybrid poplar establishment and growth at eight sites across the Eastern Townships (Truax et al., 2012). A subset of six sites with the best poplar canopy development was used for the present study. The site characteristics are summarized in Table 4. The plantations were all established on privately-owned fields that had been used for either grazing or crops, and subsequently abandoned. The vegetation before planting ranged from herbaceous to mixed herbaceous - shrub communities. Sites were prepared for planting in the fall of 1999 by ploughing and disking, and re-sprouting or germinating vegetation was eliminated in June 2000 by an application of glyphosate herbicide over the entire plantation area. Following this treatment, 2 m long rooted cuttings of nine different hybrid poplar clones were planted by hand at each site at a stem density of 833 ha^-1^ (4 m between rows and 3 m between stems along the row). Glyphosate was again applied in June 2001, but only between the rows.

After 8 seasons of growth, a wood volume production gradient had emerged across the sites (Truax et al. 2012). At certain sites, the plantations showed exceptional growth, while at others they showed average or poor growth. The majority of these plantations had, however, developed a closed canopy. In this study, understory native herbaceous plants were planted in the six most successful of these plantations.

### Experimental design

Each plantation contains three replicate 12 m × 12 m plots of each of nine hybrid poplar clones in a randomized block design. For this study we used only two of these clones, namely clone 915303, a hybrid of *Populus maximowiczii* and *P. balsamifera* (M × B), and clone 131, a hybrid of *P. deltoides* and *P. nigra* (D × N), both developed in Quebec. Clone 3333, another D × N hybrid, was used in place of clone 131 where the latter showed high damage. At each site, three 12 m × 12 m woodlot plots were selected within a secondary forest stand close to the plantation. At the BED and HAM sites, the woodlot plots were located in two different forest stands, both close to the plantations. These forested areas had regenerated naturally after either the harvest of a previous stand, or the abandonment of agriculture. The age of each stand was estimated based on cores taken from a sample of the largest trees and interviews with landowners. The vegetation and environmental characteristics of these woodlot plots are described in Boothroyd-Roberts et al. (2013) (see also Table 4 for dominant tree species). Thus, the present study consisted of three woodlot plots per site and three plots per site in the M × B poplar plantations and in the D × N poplar plantations, for a total of 54 plots (6 sites × 3 stand types × 3 replicates). Four woodlot plots (three from the LAP site and one from the HAM site) were subsequently eliminated from the study due to damage from fallen trees or crushed deer exclusion cages.

We established experimental plantations of understory native herbaceous plants within each of the plots described above. We chose four species typical of mature broadleaf forests in the region, with conservation value and economic value, which could potentially serve as non-timber forest products (NTFPs). These are: *Asarum canadense* L.*, Maianthemum racemosum* (L.) Link (syn. *Smilacina racemosa*), *Sanguinaria canadensis* L. and *Trillium grandiflorum* (Michx.) Salisb. All of these species, apart from *Maianthemum*, are officially designated as vulnerable species in Quebec, due to the threat of overexploitation of wild populations. *Asarum* is sought because of the essential oils contained in its rhizomes, while *Sanguinaria* is sought for its rhizome containing sanguinarine, a powerful alkaloid with known medicinal uses. *Trillium* and *Asarum* are both valued for their ornamental value in shaded gardens. *Maianthemum* is also used for horticulture to a lesser extent. No natural populations of any of the transplanted species were present in any plot; however, a patch of *Asarum* was observed near one of the plots in the Bromptonville woodlot.

### Transplantation of understory herbs

Small plants (well-established seedlings) of these four species were purchased from Horticulture Indigo, a specialized nursery that cultivates native plants from seed, thus avoiding an impact on wild populations. This nursery is located in Melbourne, at the centre of our study area, avoiding any unnecessary transportation time that could compromise plant quality. We chose to use plants because introductions of forest perennial herbs have been shown to be more successful from transplants than from seed (Francis and Morton 1995, Primack 1996). We planted *Asarum* and *Maianthemum* in all 54 plots over the six sites. Due to the limited availability of plants, we planted *Sanguinaria* and *Trillium* at only three of the sites (27 plots; BED, BRO, LAP). In each plot, we planted 10 individuals (*Trillium* and *Maianthemum*) or 10 clumps (*Sanguinaria* and *Asarum*) of each species. Occasionally, 11 or 12 individuals of *Trillium* or *Maianthemum* were planted in cases in which it was impossible to separate two that had grown together. In total, 1636 plants were planted. The plants were introduced into small rectangular understory plantations from which all understory vegetation, large surface roots (to 15 cm depth), and litter were removed by hand. These understory plantations consisted of two rows per species and five plants per row with a spacing of 20 cm between plants and 20 cm between the rows (total areas of 120 cm × 180 cm for four species, and 120 cm × 100 cm for two species). The experimental plants were transplanted between May 25, 2009 and June 4, 2009. No weeding has been done since transplantation, but we protected the plants from larger herbivores (mostly white-tailed deer) with exclusion cages.

### Understory herb measurements

The size of all transplanted plants was recorded following transplantation, between June 10 and 18, 2009, and again in the two following springs (2010 and 2011). *Sanguinaria* flowers were counted on April 22, 2010 and May 5, 2011 at the Bedford site; April 29, 2010 and May 7, 2011 at the Bromptonville site; and May 30, 2010 and May 14, 2011 at the La Patrie site. All remaining counts and measurements were taken between May 21 and June 3, 2010 and June 6 and June 9, 2011. *Sanguinaria* flowers were not counted the first year because the flowering season had finished before the time of transplantation.

For each species, we used different measurements, appropriate for the morphology and growth patterns of each, as proxies for the total biomass of the species in the plot. For *Asarum* and *Sanguinaria*, we measured the sum of the number of leaves and the number of flowers in each plot, while for *Maianthemum* and *Trillium*, we measured the sum of the leaf length of all leaves in the plot. *Maianthemum* and *Trillium* flowers were also counted.

### Environmental characteristics

The environment within each plot was characterized during the 2009 growing season by its soil chemical and physical properties, stand basal area, light availability in the understory, and leaf litter biomass. Detailed methods and results of these measurements are presented in a previous article (Boothroyd-Roberts et al. 2013). Briefly, we collected five soil samples distributed systematically within each plot for chemical analysis, which were then combined into one composite. Samples were taken from the mineral soil at a depth of between 5 cm and 10 cm, corresponding to the principal rooting zone of herbaceous plants. We measured pH in a soil-water suspension. The available potassium (K), calcium (Ca), and magnesium (Mg) contents were determined through extraction with BaCl_2_ and detection by atomic absorption. The extractable phosphorus (P) content was measured using the Bray-2 method (Bray and Kurtz 1945) (modified by F. Lambert). The total nitrogen (N) and total carbon (C) were measured using dry combustion, high-temperature reduction of the combustion products, and thermo-conductometric detection. Soil moisture at a depth of 10 cm was sampled twice, in June and August, corresponding to dry periods with no significant rainfall events in the 48 hours prior, and measured gravimetrically after oven drying. Light availability at a height of 90 cm was measured using a digital hemispheric photograph, taken at the centre of the plot, to determine canopy openness and the average light received during the growing season. Leaf litter was collected after almost all the leaves had fallen from every tree from a 50 cm × 50 cm microplot and was subsequently dried and weighed.

### Analyses

The responses of each understory herb species were analyzed using separate 2-way analysis of variance tests (ANOVAs) with stand type (M×B poplar plantation vs. D×N poplar plantation vs. woodlot), site and their interaction as fixed factors. The LAP site was excluded from ANOVAs because data was missing from all woodlot plots from this site. This left two remaining sites for *Sanguinaria* and *Trillium,* and five sites for *Asarum* and *Maianthemum*. The experimental unit was the plot (n = 3 per cell). All response measures were subjected to square-root transformations to improve the normality of ANOVA residuals and homoscedascicity; for presentation purposes the raw data are used in figures and tables. ANOVAs were done using the JMP software package (SAS Institute, Cary, NC).

To explore the relative influence of different biotic and abiotic environmental variables on the response of transplanted plants, we fit a series of linear mixed-effect regression models, which we then used for model selection and multi-model inference. Each understory herb response was fit separately and all response variables were square-root transformed prior to analysis. The replication level for all models was the plot, using all plots except for the four woodlot plots with missing data (n = 50 for *Asarum* and *Maianthemum*; n = 24 for *Sanguinaria* and *Trillium*). We began with an initial set of eight environmental variables as fixed factors and site as a random factor to account for the spatial clustering of plots. Site was not considered a fixed factor for this analysis since the question of interest was the relative effects of the different environmental variables rather than site effects. The initial set of environmental variables consisted of elevation, June soil moisture, soil C:N ratio, soil Ca, soil Mg, soil P, leaf litter biomass and availability of diffuse light. August soil moisture, soil C, soil N, soil K, soil pH and availability of direct light were excluded from the analyses because they were highly correlated with one of the selected variables (r > 0.6). For each response variable, we selected a single best model from all possible subsets of the initial set of environmental variables, using maximum likelihood and Aikaike’s Information Criterion for small samples (AICc: Burnham and Anderson 2002). We calculated a marginal R^2^ for these best models as an estimate of the variance explained by the fixed factors in the model (Nakagawa and Schielzeth 2013). Because there is often uncertainty in choosing the best model, multi-model inference was also done, using all models within 4 AICc of the best model. A relative importance value was calculated for each environmental variable as the sum of the Aikaike weights of all models in which the variable was included (Aikaike weights quantify the probability that a given model is the best model). Model-averaged regression coefficients were also calculated. Initial mixed-effects models were fit using the lme4 package (Bates et al. 2013) in R software (R Development Core Team 2011) and the MuMIn package (Barton 2013) was used for model selection and multimodel inference.

## References

[CR1] Albrecht MA, McCarthy BC (2009). Seedling establishment shapes the distribution of shade-adapted forest herbs across a topographical moisture gradient. J Ecol.

[CR2] Aubin I, Messier C, Bouchard A (2008). Can plantations develop understory biological and physical attributes of naturally regenerated forests?. Biol Conserv.

[CR3] Baeten L, Hermy M, Verheyen K (2009). Environmental limitation contributes to the differential colonization capacity of two forest herbs. J Veg Sci.

[CR4] Barton K (2013). MuMIn: multi-model inference. R package version 1.9.5.

[CR5] Bates D, Maechler M, Bolker B (2013). lme4: linear mixed-effects models using S4 classes.R package version 0.999999-2.

[CR6] Bélanger L, Grenier M (2002). Agriculture intensification and forest fragmentation in the St. Lawrence valley, Québec, Canada. Landsc Ecol.

[CR7] Bierzychudek P (1982). Life histories and demography of shade-tolerant temperate forest herbs: a review. New Phytol.

[CR8] Boothroyd-Roberts K, Gagnon D, Truax B (2013). Can hybrid poplar plantations accelerate the restoration of forest understory attributes on abandoned fields?. For Ecol Manag.

[CR9] Bray RH, Kurtz LT (1945). Determination of Total, Organic, and Available Forms of Phosphorus in Soils. Soil Sci.

[CR10] Bremer LL, Farley KA (2010). Does plantation forestry restore biodiversity or create green deserts? A synthesis of the effects of land-use transitions on plant species richness. Biodivers Conserv.

[CR11] Brockerhoff EG, Jactel H, Parrotta JA, Quine CP, Sayer J (2008). Plantation forests and biodiversity: oxymoron or opportunity?. Biodivers Conserv.

[CR12] Brunet J, De Frenne P, Holmstrom E, Mayr ML (2012). Life-history traits explain rapid colonization of young post-agricultural forests by understory herbs. For Ecol Manag.

[CR13] Bruun HH, Valtinat K, Kollmann J, Brunet J (2010). Post-dispersal seed predation of woody forest species limits recolonization of forest plantations on ex-arable land. Preslia.

[CR14] Burkhart EP, Jacobson MG (2009). Transitioning from wild collection to forest cultivation of indigenous medicinal forest plants in eastern North America is constrained by lack of profitability. Agrofor Systems.

[CR15] Burnham KP, Anderson D (2002). Model selection and multi-model inference.

[CR16] Case FWJ (2002). Trillium grandiflorum.

[CR17] Charron D, Gagnon D (1991). The demography of northern populations of *Panax quinquefolium* (American Ginseng). J Ecol.

[CR18] De Keersmaeker L, Martens L, Verheyen K, Hermy M, Schrijver AD, Lust N (2004). Impact of soil fertility and insolation on diversity of herbaceous woodland species colonizing afforestations in Muizen forest (Belgium). For Ecol Manag.

[CR19] Domon G, Bouchard A (2007). The landscape history of Godmanchester (Quebec, Canada): Two centuries of shifting relationships between anthropic and biophysical factors. Landsc Ecol.

[CR20] Endels P, Adriaens D, Verheyen K, Hermy M (2004). Population structure and adult plant performance of forest herbs in three contrasting habitats. Ecography.

[CR21] Fischer D, Hart S, Rehill B, Lindroth R, Keim P, Whitham T (2006). Do high-tannin leaves require more roots?. Oecol.

[CR22] Flinn KM, Vellend M (2005). Recovery of forest plant communities in post-agricultural landscapes. Front Ecol Environ.

[CR23] Flinn KM, Vellend M, Marks PL (2005). Environmental causes and consequences of forest clearance and agricultural abandonment in central New York, USA. J Biogeogr.

[CR24] Fortier J, Gagnon D, Truax B, Lambert F (2010). Biomass and volume yield after 6 years in multiclonal hybrid poplar riparian buffer strips. Biomass Bioenergy.

[CR25] Fortier J, Gagnon D, Truax B, Lambert F (2011). Understory plant diversity and biomass in hybrid poplar riparian buffer strips in pastures. New For.

[CR26] Fortier J, Truax B, Gagnon D, Lambert F (2012). Hybrid poplar yields in Québec: implications for a sustainable forest zoning management system. For Chron.

[CR27] Foster DR (1992). Land-use history (1730-1990) and vegetation dynamics in central New England, USA. J Ecol.

[CR28] Francis JL, Morton AJ, Urbanska KM, Grodzinska K (1995). Restoring the woodland field layer in young plantations and new woodlands p. Restoration Ecology in Europe.

[CR29] Frère Marie-Victorin ÉC, Brouillet L, Hay SG, Goulet I (1995). Flore Laurentienne.

[CR30] Gagnon D, Bouchard A (1981). La végétation de l'escarpement d'Eardley, Parc de la Gatineau, Québec. Can J Bot.

[CR31] Gardiner ES, Stanturf JA, Schweitzer CJ (2004). An afforestation system for restoring bottomland hardwood forests: biomass accumulation of nuttall oak seedlings interplanted beneath eastern cottonwood. Restor Ecol.

[CR32] Gauthier S, Gagnon D (1990). La végétation des contreforts des Laurentides: une analyse des gradients écologiques et du niveau successionnel des communautés. Can J Bot.

[CR33] Glitzenstein JS, Canham CD, McDonnell MJ, Streng DR (1990). Effects of environment and land-use history on upland forests of the Cary arboretum, Hudson Valley, New York. Bull Torrey Bot Club.

[CR34] Godefroid S, Vanderborght T (2011). Plant reintroductions: the need for a global database. Biodivers Conserv.

[CR35] Hunter MD (2001). Out of sight, out of mind: the impacts of root-feeding insects in natural and managed systems. Agric For Entomol.

[CR36] Kiger RW (1997). Sanguinaria canadensis.

[CR37] LaFrankie JV (2002). Maianthemum racemosum.

[CR38] Lamoureux G (2002). Flore printanière.

[CR39] Le Houérou HN (1993). Land degradation in Mediterranean Europe: can agroforestry be a part of the solution? a prospective review. Agrofor Syst.

[CR40] Liang Y, Stehlik I (2009). Relationship between shade and herbivory in *Asarum canadense* (Wild Ginger). Univ Tor J Undergrad Life Sci.

[CR41] Lubbers AE, Lechowicz MJ (1989). Effects of leaf removal on reproductions vs. belowground storage in *Trillium grandiflorum*. Ecol.

[CR42] Marchand PP, Masse S (2007). Boisement et agroforesterie en courtes rotations en territoire privé au Québec: Examen des lois, règlements, politiques et programmes. Ressources naturelles Canada, Service canadien des forêts, Centre de foresterie des Laurentides.

[CR43] (2010). Plantes menacées ou vulnérables au Québec.

[CR44] Mottl LM, Mabry CM, Farrar DR (2006). Seven-year survival of perennial herbaceous transplants in temperate woodland restoration. Restor Ecol.

[CR45] Nakagawa S, Schielzeth H (2013). A general and simple method for obtaining R^2^ from generalized linear mixed-effects models. Method Ecol Evol.

[CR46] Nantel P, Gagnon D, Nault A (1996). Population viability analysis of American ginseng and wild leek harvested in stochastic environments. Conserv Biol.

[CR47] Nault A, Gagnon D (1993). Ramet demography of *Allium tricoccum*, a spring ephemeral, perennial forest herb. J Ecol.

[CR48] Peterken GF (2000). Rebuilding networks of forest habitats in lowland England. Landsc Res.

[CR49] Poyatos R, Latron J, Llorens P (2003). Land use and land cover change after agricultural abandonment: the case of a Mediterranean mountain area (Catalan Pre-Pyrenees). Mt Res Dev.

[CR50] Primack RB, Falk DA, Millar CI, Olwell M (1996). Lessons from ecological theory: dispersal, establishment and population structure *p* 209*–*233. Restoring diversity: strategies for reintroduction of endangered plants.

[CR51] (2005). Trille blanc.

[CR52] (2011). R: a language and environment for statistical computing.

[CR53] Roura-Pascual N, Pons P, Etienne M, Lambert B (2005). Transformation of a rural landscape in the Eastern Pyrenees between 1953 and 2000. Mt Res Dev.

[CR54] Ruhren S, Handel SN (2003). Herbivory constrains survival, reproduction and mutualisms when restoring nine temperate forest herbs. J Torrey Bot Soc.

[CR55] Schimel JP, Cates RG, Ruess R (1998). The role of balsam poplar secondary chemicals in controlling soil nutrient dynamics through succession in the Alaskan taiga. Biogeochem.

[CR56] Singleton R, Gardescu S, Marks PL, Geber MA (2001). Forest herb colonization of postagricultural forests in central New York State, USA. J Ecol.

[CR57] Soo T, Tullus A, Tullus H, Roosaluste E (2009). Floristic diversity responses in young hybrid aspen plantations to land-use history and site preparation treatments. For Ecol Manag.

[CR58] St-Jacques C, Gagnon D (1988). La végétation forestière du secteur nord-ouest de la vallée du St-Laurent, Québec. Can J Bot.

[CR59] Truax B, Gagnon D, Fortier J, Lambert F (2012). Yield in 8 year-old hybrid poplar plantations on abandoned farmland along climatic and soil fertility gradients. For Ecol Manag.

[CR60] Vasseur L, Gagnon D (1994). Survival and growth of *Allium tricoccum* Ait. transplants in different habitats. Biol Conserv.

[CR61] Whigham DF (2004). Ecology of woodland herbs in temperate deciduous forests. Annu Rev Ecol Evol Syst.

[CR62] Whittemore AT, Mesler MR, Lu KL (1997). Asarum canadense.

